# Sustainable Wheat Production and Food Security of Domestic Wheat in Tajikistan: Implications of Seed Health and Protein Quality

**DOI:** 10.3390/ijerph18115751

**Published:** 2021-05-27

**Authors:** Bahromiddin Husenov, Siham Asaad, Hafiz Muminjanov, Larisa Garkava-Gustavsson, Eva Johansson

**Affiliations:** 1 Department of Plant Breeding, The Swedish University of Agricultural Sciences, Box 101, SE-230 53 Alnarp, Sweden; Larisa.Gustavsson@slu.se (L.G.-G.); Eva.Johansson@slu.se (E.J.); 2 Agronomy Faculty, Tajik Agrarian University Named after Sh. Shohtemur, 146, Rudaki ave., Dushanbe 7340003, Tajikistan; 3 International Centre for Agricultural Research in the Dry Areas (ICARDA), Dalia Building 2nd Floor, Bashir El Kassar Street, Verdum, Beirut 1108-2010, Lebanon; siham531@yahoo.com; 4 Food and Agriculture Organization of the UN (FAO), Viale delle Terme di Caracalla, 00153 Rome, Italy; hafiz.muminjanov@fao.org

**Keywords:** common bunt, crop management, environment protection, field survey, food security, seed-borne disease, wheat protein

## Abstract

Staple crop yield, quality and sustainable production are critical for domestic food security in developing countries. In Tajikistan, both seed-borne diseases and protein quality impair the yield and the quality of the major staple crop, wheat. Here, we used a detailed two-year survey of fields on 21 wheat-producing farms in Tajikistan, combined with lab analyses on seed health and protein quality, to investigate the presence of seed-borne diseases and bread-making quality in Tajik wheat. Seed samples were collected for the analysis of: (i) the presence of common bunt (*Tilletia* spp.) using the centrifuge wash test, (ii) the major pathogenic fungi on/in the seed using the agar plate test and (iii) the protein amount and size distribution using size-exclusion high-performance liquid chromatography (SE-HPLC). Field occurrence of common bunt and loose smut was generally low (3 farms in year one (14%) showed common bunt occurrence), but the presence of fungi was observed microscopically on most seed samples (on seeds from 19 out of 21 farms = 91%). *Tilletia laevis* was the dominant agent in common bunt (present in 19 farms compared to *T. tritici* present in 6 farms). Altogether, 18 different fungi were identified from seed samples by microscopy. Protein composition, measured with high-performance liquid chromatography as protein amount and size distribution (known to correlate with bread-making quality), differed significantly between samples from different farms and years, although the farm type and land elevation of the farm were not the determinants of the protein composition. The presence of certain fungi on the seed correlated significantly with the protein quality and could then have an impact on the bread-making quality of the Tajik wheat. The presence of seed-borne diseases, a low protein content and weak gluten were the characteristics of the majority of the grain samples, mostly irrespective of farm type and farmer’s knowledge. For sustainable development of the Tajik farming systems, and to strengthen the food security of the country, the knowledge of Tajik farmers needs to be increased independently of farm type; in general, plant breeding is required and certified seeds need to be made available throughout the country.

## 1. Introduction

Wheat is a staple food crop, globally contributing 20% of the calories and proteins to the human diet [1]. Thus, wheat is crucial for food supply and security in many countries around the world, and it is a critical part of the daily diet in West and Central Asia, and North Africa [2]. In Tajikistan, wheat contributes significantly to food security through a variety of bread products made and in the preparation of a dozen types of different meals in various regions of the country [3].

According to the World Food Program of the United Nations (WFP), a high proportion (30%) of the Tajik population is undernourished and about half of the population live on less then USD 1 a day [4]. Similarly, the Food and Agriculture Organization of the United Nations (FAO) places Tajikistan among the countries where development goals targeting hunger are progressing slowly and where the prevalence of undernourishment is high (33%) [5]. The ongoing COVID-19 pandemic may lead to significant increases in food prices, especially of wheat as a key product, and poverty levels in developing countries, including Tajikistan, may rise again [6]. Food insecurity and poverty are the major reasons for several serious public health issues [7]. Hence, the first important action to prevent severe health issues in developing countries like Tajikistan is to improve access for people to food, and especially to the staples. Thus, to ensure future food security in Tajikistan, sufficient wheat production in terms of quantity and quality is essential, together with sustainability in production to secure the environment.

Wheat production and yield of the crop are determined by a number of factors related to the choice of cultivar, the growing environment and cropping conditions. In Tajikistan, foliar diseases, poor seed quality with limited use of certified seeds, lack of appropriate crop management and weed control taking biodiversity into consideration, low availability of superior varieties to farmers, lack of financial investment and low level of farming knowledge among wheat farmers have been identified as major constraints on domestic wheat production and yield [8,9,10]. Currently, sustainable weed control and certified seeds are two measures used to a limited extent, but with large opportunities to sustainably effect the wheat production and yield [10]. Until now, some work has been carried out to improve seed quality through breeding for resistance to the main foliar diseases [9,11], while both weed management and seed-borne diseases have been handled limitedly. In a recent paper, weed management strategies that simultaneously focused on not hampering the extensive weed biodiversity in Tajikistan were discussed and propositions were made [10]. Seed-borne diseases, which are transmitted to next generation of wheat through the seed [12], are a major concern for Tajik wheat production, since farmers commonly use saved seeds without proper quality testing [8,10,13]. Seeds can host a number of potentially pathogenic microorganisms [14], and non-certified seeds or seeds from unknown sources have been shown to bear significantly higher levels of seed-borne diseases [15]. Thus, to obtain high yields without the use of certified and resistant seeds would require the extensive use of chemical pesticide treatments. The major wheat seed-borne diseases are caused by fungi [14]. A broad spectrum of fungi are present on the wheat seed, but the most important are common bunt (*Tilletia tritici* (Bjerk.) Wint, *T. laevis* Kühn) and loose smut (*Ustilago tritici* (Pres.) Rostr.) [16]. Furthermore, black pointed seeds are mainly known as a result of the presence of certain fungi on the seed [10,17]. In most countries worldwide, seed-borne diseases are strictly monitored, particularly within the grain industry [18,19], as well as in seed certification and seed movement [20]. The breeding of resistant varieties contributes to sustainability in production as chemical pesticide measures can be reduced [21].

Wheat is used for a range of products of which bread is known as the major one, which is also particularly true for Tajikistan. The bread-making quality of wheat is known to depend on many factors, and in particular, the genotype, the growing environment and the management practices are of importance [22,23]. Crop management practices such as nitrogen application, irrigation, soil conditions and management have been shown to affect both grain protein concentration and protein quality [24,25,26,27]. Earlier studies have shown a negative correlation between seed-borne diseases and yield, and a negative impact of specific seed-borne diseases, e.g., common bunt, loose smut, black point and Karnal bunt, on wheat grain and flour quality [28,29,30]. Different protein fractions in wheat also affect bread-making quality [31], e.g., there is a positive correlation between percentage of sodium dodecyl sulphate (SDS)-unextractable polymeric protein in total polymeric protein (%UPP), gluten strength and bread-making quality [32,33]. In Tajikistan and other similar countries, bread-making quality is essential in a food security perspective due to the importance of the bread in the daily diet of the Tajik people [34].

The overall objective of the present study was to evaluate opportunities for sustainable wheat production with increased yield and quality to improve food security in a developing country such as Tajikistan. Thereby, the aims were to: (i) determine the magnitude, prevalence and pattern of fungi on wheat seeds and the presence of seed-borne diseases in wheat samples produced by farmers in Tajikistan; (ii) evaluate the protein quality of wheat produced by farmers in Tajikistan; and (iii) examine relationships and interactions between the presence of fungi on the seeds causing seed-borne diseases and protein quality in wheat produced by Tajik farmers. From the results on the seed-borne pathogens present and the defined bread-making quality, we elaborate on and suggest opportunities and a way forward for sustainable wheat production for food security. Furthermore, possible relationships between farm type, management, cultivation environment, quantity and quality of wheat production and the presence of fungi on the seeds and of seed-borne diseases in wheat produced by Tajik farmers were examined.

## 2. Materials and Methods

### 2.1. Field Surveillance

#### 2.1.1. Surveyed Farms

**Figure 1:**
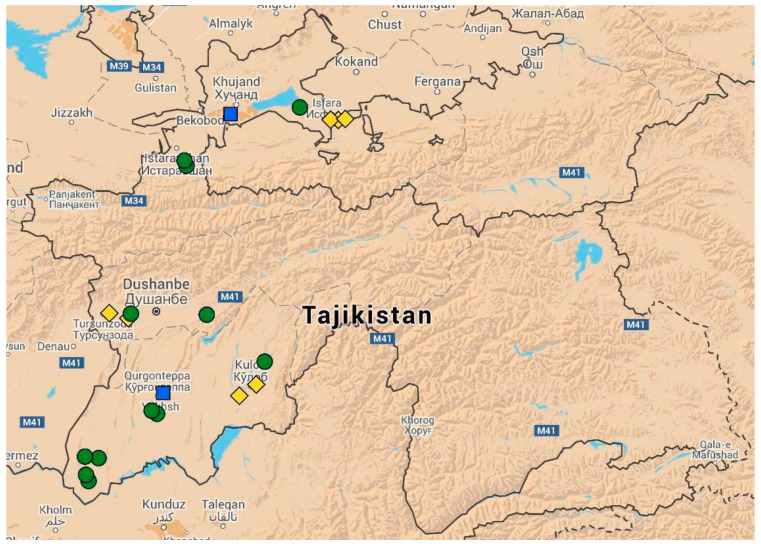
Map of Tajikistan (Google maps) showing the location of survey sites. *Dehkan* farms (DF, *n* = 13) are indicated by green circles, production cooperatives (PC, *n* = 6) by yellow diamonds and the Tajik Farming Institute (TFI, *n* = 2) by blue squares.

A total of 21 wheat-growing farms throughout Tajikistan were selected for in-depth analysis (Figure 1). In selection, the aim was to cover the main wheat-growing areas of Tajikistan and capture differences in farm elevation, cropping systems, growing conditions and farm and field size (Table 1). Farm structure in Tajikistan underwent a transition from the large-scale state farms and household plots of the Soviet era to medium and small peasant (*dehkan*) farms [34] with a low level of mechanization. The types of farms covered in the survey were *dehkan* farms (DF, *n* = 13), larger, more mechanized production cooperatives (PC, *n* = 6) based on former collective farms (*Kolkhoz*) or Soviet farms (*Sovkhoz*) and field stations of the Tajik Farming Institute (TFI, *n* = 2), which are larger and more mechanized and which receive improved seeds directly from TFI breeders for further multiplication. The survey of farms was conducted during two consecutive years, 2011 and 2012, at the flowering to dough stage of the wheat plants. Each farm was visited once per season.

#### 2.1.2. Field Survey Methodology

A questionnaire (see Table S1 in Supplementary Materials) was developed to obtain information about: (1) the farm, including farm location, management and ownership; (2) field and crop management issues, such as geographical location, access to advanced varieties and good quality seed, use of pesticides, pests and diseases managements, fertilizer use and other relevant information; and (3) crop health status, with particular attention to presence of major seed-borne diseases, namely common bunt and loose smut, and the presence of other diseases, pests and weed plants, using rating scales described in Husenov et al. [10].

The occurrence of seed-borne diseases was assessed as the percentage of diseased plants. Fields were visually inspected alongside the whole field, and if the presence of common bunt or loose smut was noted, 3–6 samples, each consisting of 100 plants, were collected at spots randomly spread over the field. These samples were visually inspected, and the incidence of disease was calculated as the percentage of infected plants in the total number of plants per sample. The mean incidence in the field was then calculated.

The presence of dominant weed species per square meter was assessed and weed density in the field was classified as low (<20%), medium (20–40%) and high (>40%).

### 2.2. Seed Health and Protein Assessments

#### 2.2.1. Grain Sampling

Grain samples were collected from surveyed fields at harvest and used for analyses of seed health and protein quality and quantity. Each farm harvested their wheat fields in full maturity time and all grains from a surveyed field were bulked in a separate lot. Sampling was carried out using national seed sampling standard methods employed also in seed quality testing following the International Seed Testing Association (ISTA) rules [35]. Primary samples were taken from the bulk grain in accordance with ISTA rules and these samples were pooled to obtain a composite sample. A 200 g subsample was taken from this composite sample in a paper envelope for further laboratory analyses. All tests to differentiate seed-borne pathogens on the wheat grain were carried out at the Swedish University of Agricultural Sciences (SLU), in close contact and collaboration with the Seed Health Laboratory at the International Center for Agricultural Research in the Dry Areas (ICARDA), Syria, applying well-developed and previously tested and described methods [36]. The seed samples were shipped after collection to Sweden via DHL express post service (www.dhl.com (accessed on 20 May 2021) and kept in the seed storage facility of SLU, Alnarp at +4 °C until the start of tests.

#### 2.2.2. Centrifuge Wash Test

The centrifuge wash test (CWT) was carried out for detection of teliospores of *Tilletia* spp., the main cause of common bunt, and spores of some other fungal pathogens attached to the seed surface [37]. Following the procedure described in [36], 25 g of the seed samples was soaked, shaken for one minute, and thereafter kept for 12–16 h at room temperature (RT). The suspension was then poured into separate tubes and centrifuged at 2000 rpm for 10 min. The sediment obtained after centrifugation was examined under a compound microscope (200× magnification) for *Tilletia* spp. and other fungi, as described by Mathur and Kongsdal [37]. For differentiation of teliospores of *Tilletia* spp., specifically *T. laevis* and *T. tritici*, the descriptions given by Mathur and Kongsdal [37], and Wilcoxson and Saari [16] were used. When a more precise check was needed, higher magnification was used. Seed samples were also visually inspected for presence of bunted ball seed or other impurities.

#### 2.2.3. Agar Plate Test

Prior to plating on the agar medium, all wheat kernels were visually evaluated for the presence of undesired inert materials, i.e., plant debris, weed seeds and other impurities. The presence of black points (black-brown pigmentation in the grain coat overlaying the embryo and scutellum [17]) was also visually assessed and the percentage of pigmented kernels was calculated. Agar plate tests were applied to find major fungal pathogens on the seed surface using the procedure described by Diekmann [19]. For this, 10 g of grain was taken from the original sample and surface sterilized with 5% sodium hypochlorite (NaOCl) for 2–3 min. The samples were then dried on filter paper and a total of 130 seeds from each sample were placed on petri dishes (10 dishes, each with 13 seeds) of potato dextrose agar (PDA) medium. The PDA used in this study was from Sigma-Aldrich and consisted of agar (15 g/L), dextrose (20 g/L) and potato extract (4 g/L) (https://www.sigmaaldrich.com/catalog/product/sial/70139. Accessed on 20 May 2021). Each dish was considered a separate replicate and all were incubated at 20 °C for 8–10 days under 14 h darkness and 10 h near ultraviolet (UV) light (to stimulate sporulation) [19]. Fungal colonies were then examined by stereomicroscope and compound microscope under different magnifications, and identified on the basis of illustrated guides compiled by the Barnett and Hunter [38], and Dugan [39], in collaboration with the seed health lab at ICARDA, Syria.

#### 2.2.4. SE-HPLC

**Figure 2:**
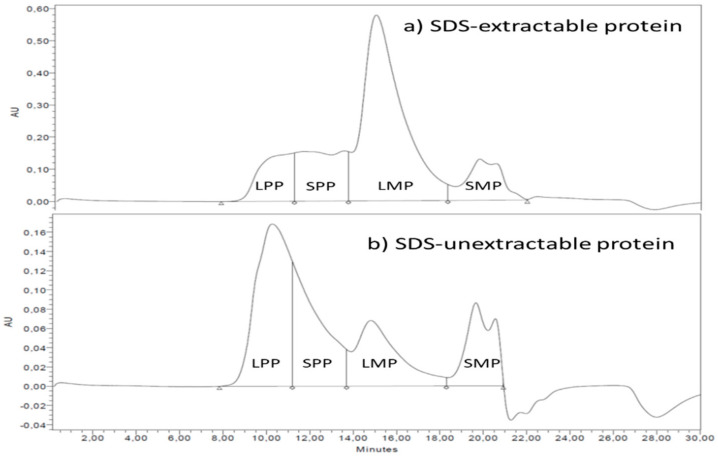
A representative chromatogram (from F11) showing SDS-extractable and SDS-unextractable proteins divided into the four parts containing large polymeric proteins (LPP), small polymeric proteins (SPP), large monomeric proteins (LMP) and small monomeric proteins (SMP).

The amount and size distribution of polymeric and monomeric proteins were determined by size-exclusion high-performance liquid chromatography (SE-HPLC) in a two-step extraction procedure according to [33] with modifications by Johansson et al. [31]. A representative chromatogram is included as Figure 2.

Samples were extracted and run in triplicates. SDS-extractable proteins were extracted in the first step after which SDS-unextractable proteins were extracted by sonication. SE-HPLC analyses were carried out with the Waters HPLC system (Milford, NH, USA) with a Phenomenex BIOSEP SEC-4000 column (Torrance, CA, USA). The total relative amount of SDS-extractable (TOTE) and SDS-unextractable (TOTU) proteins were determined from the area under the chromatogram [40]. The chromatogram was divided into four parts based on molecular size: large polymeric proteins (LPP), small polymeric proteins (SPP), large monomeric proteins (LMP) and small monomeric proteins (SMP) [41]. From these parts, the %UPP and %Large UPP were calculated [42].

### 2.3. Statistical Analyses

The frequency of infection (frequency of evaluated samples with presence of the fungi evaluated [15,43] in samples collected (tested)) was calculated as described below:

$$\mathrm{Frequency}\;\mathrm{of}\;\mathrm{infection},\;\%=\frac{\mathrm{Number}\;\mathrm{of}\;\mathrm{samples}\;\mathrm{with}\;\mathrm{infection}\;}{\mathrm{Number}\;\mathrm{of}\;\mathrm{samples}\;\mathrm{collected}\;}\times 100$$

Microsoft Excel, Statistical Analysis Systems, SAS [44], and the statistical package Minitab v17 [45] were used for statistical analyses. Minitab was applied to calculate the analyses of variance (ANOVA) for the dependent variables (frequency of infection with different *Tilletia* species and protein fractions), using factors (year, farm) as independent variables. Following ANOVA, the mean frequency of infection and protein fractions were calculated on samples from different farms, and Tukey’s post hoc test at the significance level *p* < 0.05 was used to compare means.

In order to determine and visualize the variation in protein composition between farm types and elevation categories, principal component analysis (PCA; SAS 2004) was applied to present the variables in an orthogonal data matrix, similar to what has been done in previous studies [46,47,48]. Relationships between the presence of seed-borne pathogens and protein quality parameters were also evaluated by PCA, with variation in the independent factors (farm type, elevation) visualized in a score plot and variation in the dependent variables (protein composition, diseases) visualized in a loading plot using the method described by [49]. The Spearman rank correlation was carried out to check for correlations between the protein composition and the presence of seed-borne diseases in samples.

## 3. Results

### 3.1. Major Findings from Field Inspections and Interviews

#### 3.1.1. Wheat Management in the Farms

The surveyed farms of the PC type (6/21; Table 1) had relatively better production conditions and access to inputs and machineries than the DF and TFI types. Among the 21 farms studied, 11 produced wheat grain for food purposes only, while the remaining 10 farms produced wheat to be used as a grain for both food and seeds for planting. A total of 76% (16/21) of the farmers had knowledge of the wheat varieties they were growing (variety name and origin; Table S2 in Supplementary Materials), while the remaining farmers did not. All farms surveyed planted their wheat in September–November and harvested the grain in June–July of the next year. All PC-type farms reported that wheat was sown using a drill, while only two DF farms (F10 and F16) used a drill. The remaining 11 DF farms and 2 TFI farms hand-broadcast their seeds. Four PCs, two DFs and one TFI (F13) reported use of seed treatment before planting, while the other farms did not use any chemical seed treatment. Fungicides with the trade names Dividend (active ingredient (a.i.): difeneconazole), Vitavax (a.i.: carboxin and thiram) and Raxil (a.i.: tebuconazole) were reported to be used for seed treatment. None of the farms surveyed reported use of chemical pesticides to control pests and diseases during crop growth. Additionally, 3 farms out of 21 reported the one time use of herbicide application to control weeds on one occasion during the cropping season: F12 used the herbicide Gezagard (a.i.: prometryn) and at F15 and F16 the herbicide Granstar (a.i. tribenuron-methyl) was used. Two farms practiced rainfed cropping, while the remainder irrigated their wheat at least two times during the growing season. Crop rotation was routinely applied by the PC- and TFI-type farms, while some DFs did not apply any crop rotation. The preceding crops reported included cotton, potato, maize and watermelon. All farms reported the use of nitrogen fertilizers at least once during the season. Depending on resources and availability, the N dose applied ranged from 30 to 180 kg/ha. No additional fertilizers were applied on the 21 farms surveyed (Table S2).

#### 3.1.2. Field Occurrence of Common Bunt

Common bunt incidence was high in three fields during the first study year (2011). The highest incidence (>50%) was found on farm F3 (DF type), an intermediate level (25%) on farm F4 (DF type) and the lowest incidence (5%) on farm F13 (TFI type). No common bunt occurrence was observed during year two (2012). The farm F3 with high incidence in year one, which lost 50% of grain yield in that season due to common bunt, reported growing a different variety in year two (2012).

#### 3.1.3. Occurrence of Loose Smut

Loose smut was observed only on farm F7 (PC type) in year one (2011) and only on farms F10 (DF type), F13 (TFI type) and F18 (PC type) in year two (2012), with less than 1% infection in all three cases.

#### 3.1.4. Additional Findings in Field Surveys

A number of other diseases of different rates were also observed in the field surveys. Among the foliar diseases, tan spot and leaf spot were observed frequently. Powdery mildew, *Septoria tritici*, leaf rust and a few other diseases were also observed (Table S3 in Supplementary Materials).

Weeds were found at a medium-to-high density irrespective of farm type. Higher weed density was observed in year one (2011) than in year two (2012; Table S3). The three farms (F12, F15 and F16) that reported a single application of herbicides during the growing season still had medium-to-high weed density.

### 3.2. Seed-Borne Fungi of Farmers Wheat

#### 3.2.1. Common Bunt Causes Detected by CWT

The presence of common bunt teliospores was detected by CWT in wheat samples from all farms except F5 and F15 (both PC type) over the two-year study period (Table 2). In year one (2011), samples collected from farm F10 (DF type) were also free of *Tilletia* spores, while in year two (2012), samples from farms F5, F6, F15, F17 and F20 were free of spores. The major agent in common bunt was identified as *Tilletia laevis*, while *T. tritici* was only present on five farms in the first year (2011) and three farms in the second (2012; Table 2). Among farms, highly significant differences (*p* < 0.001) were found for frequency of both *T. laevis* and *T. tritici*. No significant difference (*p* = 0.62) in frequency of *T. laevis* was found between the two study years, while *T. tritici* was significantly more frequently found in year one (2011) than in year two (2012; Table 2). ANOVA showed that *T. laevis* was significantly more frequent on DF and TFI farms than on PC-type farms.

#### 3.2.2. Major Fungi in Seed Samples

In total, 18 different species of fungi (*Acromonilla* sp., *Alternaria* spp., *Aspergillus flavus*, *Aspergillus niger*, *Bipolaris sorokiniana*, *Botrytus* spp., *Chaetomium* spp., *Cladosporium* spp., *Curvularia* spp., *Epicoccum nigrum*, *Epicoccoum purpurea*, *Fusarium graminearum*, *Fusarium* spp., *Nigrospore* spp., *Penicillium* spp., *Rhyzopus* spp., *Stemphylium* spp. and *Ulocladium* spp.) were identified in the seed samples over the two years and 21 farms. The most prevalent fungus was *Alternaria* spp., followed by Nigrospore and *Aspergillus niger* (Table 3). Other fungi were identified only rarely: *Acromonilla* spp. was found in only two samples (F1 in year one, F12 in year two) and Ulocladium spp. was found in one sample (F2 in year one). Among the fungi identified, the following are known as potential causes of seed-borne diseases: *Alternaria* spp., *Bipolaris sorokiniana*, *Stemphylium* spp., *Cladosporium* spp., *Curvularia* spp. and *Fusarium* spp. [50].

Significant differences in presence of *B. sorokiniana*, *Stemphylium* spp. and *Curvularia* spp. were noted among farm types. Higher frequencies of infection of *Stemphylium* spp. and *Curvularia* spp. were noted in DF samples than in PC and TFI samples, while the highest frequency of *B. sorokiniana* was found in samples from PC-type farms (Table 4). No significant differences in frequency of *Alternaria* spp., *Cladosporium* spp. and *Fusarium* spp. were noted among samples from different farm types (Table 4).

Observed *Fusarium* species in general were not specified to species level, but very clear symptoms of *F. graminearum* were identified in samples from farm F4 (one rep. = one petri dish of agar plate test) in year one (2011) and from farm F5 (three reps) in year two (2012).

#### 3.2.3. Black Point Pigmentation

Black point pigmentation on seeds was observed in the majority of the samples from the two years and 21 farms (Table 5). The sample from farm F4 (DF type) collected in year two (2012) was found to have a significantly higher incidence (27.8%) of black point visual seed symptoms than the other samples (Table 5). Evaluation of seeds showing black point with the agar plate method showed the presence of *Alternaria* spp. in 80.6% of samples, *Aspergillus niger* in 7.5% and *Bipolaris sorokiniana* in 2.2%. The remaining 9.7% of black point seed samples were infected with saprophytic fungi.

### 3.3. Wheat Proteins

There were significant differences in the protein fractions in wheat samples from the different farms surveyed (Table 6). ANOVA revealed highly significant variation (*p* < 0.005) between farms for all protein fractions evaluated (TOTE, TOTU, %UPP and %LargeUPP). Significant variation (*p* < 0.05) was also seen in TOTE, TOTU and %UPP between the two study years while %LargeUPP did not differ significantly between years (*p* = 0.349). High TOTE was found in samples from farms F13 and F15, while F11 and F20 showed low values. High %UPP was observed in wheat from farms F5 and F9, but low %UPP was observed in samples from F12 and F15 (Table 6).

**Figure 3:**
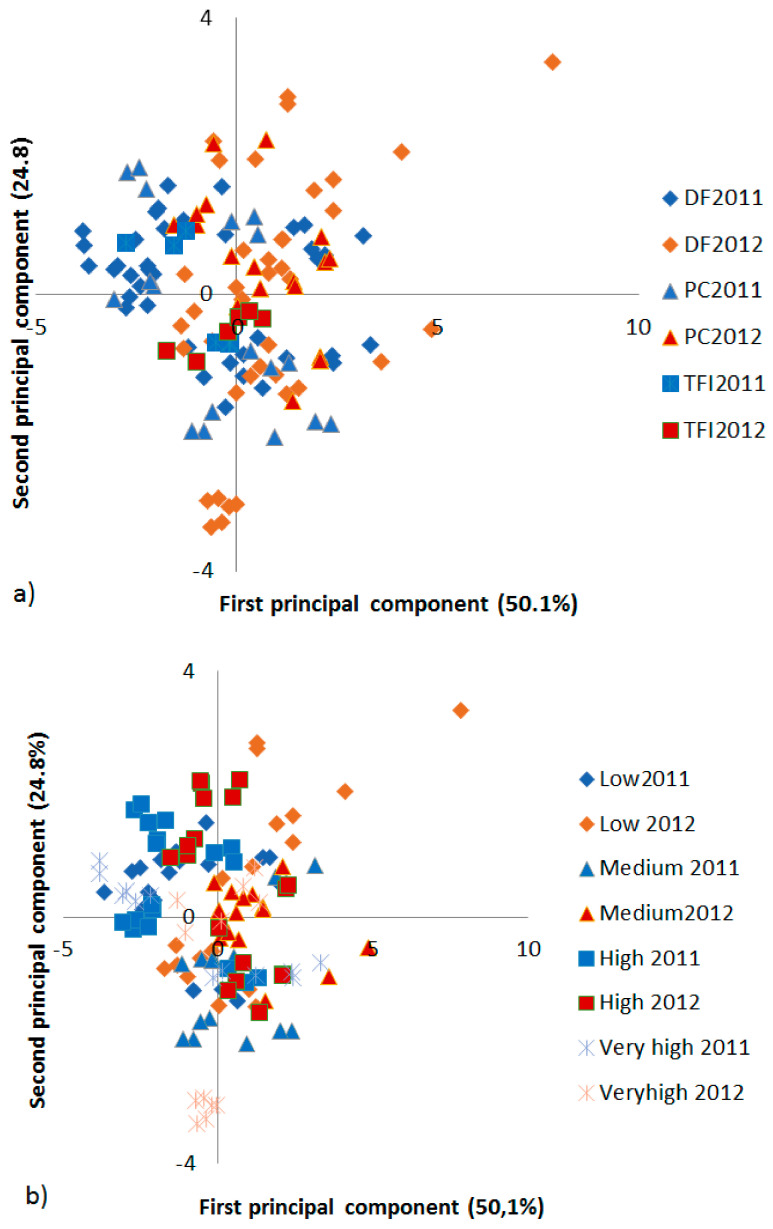
Score plot from a principal component analyses (PCA) of protein composition in wheat grain from farms (**a**) of different types (DF = *dehkan* farm; PC = production cooperative; TFI = Tajik Farming Institute farm) and grown during different years and (**b**) at different elevations.

The PCA carried out comparing the impact of farm type and land elevation on the protein composition resulted in three principal components (PCs) with eigenvalues equal to or above one (PC1 = 5.55, PC2 = 1.73 and PC3 = 1.00). The two first ones, explaining 50.1% and 24.8% of the variation, were used in score plots to visualize the results, revealing no clear correlation between the farm type (Figure 3a), nor between the elevation (Figure 3b) and PC values. Instead, samples from 2012 (red) were found with a higher PC1 value than the samples from 2011 (blue), indicating that the cultivation year had a more clear impact on the protein composition in the wheat samples than the farm type and land elevation. The loading plot (not shown) from the PCA revealed TOTE (correlated to grain protein content) with a negative PC1 (−0.31) value and %UPP (correlated to gluten strength) with a positive PC1 (0.49) value. Thus, the PCA results indicated generally higher TOTE and lower %UPP in samples from 2011 than in samples from 2012.

### 3.4. Relationship of Seed-Borne Diseases and Protein Composition of Wheat Seeds

**Figure 4:**
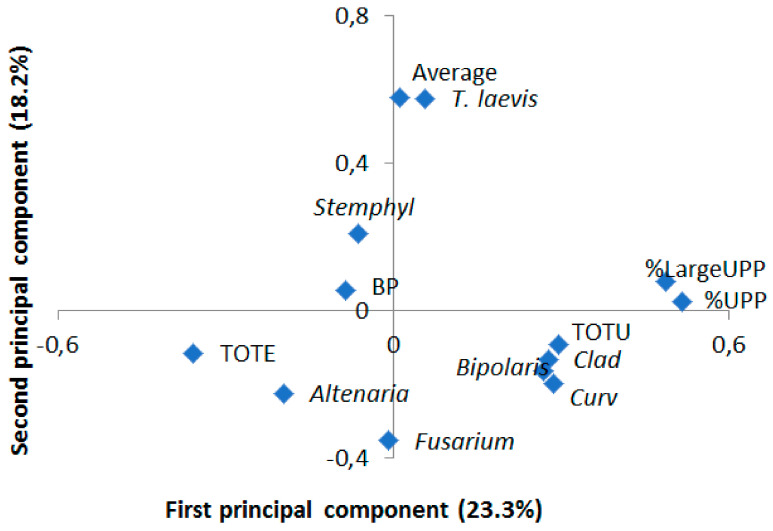
Loading plot from principal component analyses (PCA) of protein composition variables (TOTE = total SDS-extractable proteins; TOTU = total SDS-unextractable proteins; %UPP = percentage of total unextractable polymeric proteins in total polymeric proteins; %LargeUPP = percentage of large unextractable polymeric proteins in total large polymeric proteins) and various seed-borne diseases. The first principal component explained 23.3% of the variation, while the second principal component explained 18.2% of the variation.

The PCA analyses evaluating the relationship between seed-borne diseases found in the samples and their protein composition resulted in three principal components with eigenvalues above one (PC1 = 2.83, PC2 = 1.75 and PC3 = 1.36). The loading plot applying the first two analyses, explaining 23.3% and 18.2% of the variation, respectively, was used to visualize this relationship, which indicated a positive relationship between the presence of *Bipolaris sorokiniana*, *Curvularia* spp. and *Cladosporium* spp. and the %UPP and %LargeUPP, while the presence of these diseases showed a negative relationship with TOTE (Figure 4). The PCA analyses also indicated a positive relationship between the presence of *Alternaria* spp. and TOTE, while this relationship was negative with %UPP and %LargeUPP (Figure 4). Spearman rank correlation analyses showed a significant negative correlation between *Bipolaris sorokiniana* and TOTE (*p* < 0.05).

## 4. Discussion

The visual inspection of wheat fields on 21 farms in Tajikistan over two years revealed a generally low infection of common bunt (*Tilletia laevis* and *T. tritici*) and other seed-borne diseases. Despite this, grain samples from almost all farms investigated showed the presence of teliospores of both *Tilletia* species, but predominantly *T. laevis*. Moreover, the presences of other major fungi (e.g., *Alternaria* spp., *Bipolaris sorokiniana*, *Stemphylium* spp., etc.) in the seed samples were also found. The majority of samples collected from the farms showed a significant level of black point symptoms, which could be either due to susceptibility of varieties grown [51] or to late rain at the end of growing season, which enhances the disease [52]. The findings in this study indicate that a high prevalence of wheat seeds infected with seed-borne diseases and the presence of fungi may lower the quality of Tajik wheat in terms of grain protein concentration and gluten strength, thereby threatening food security for the country.

The fact that common bunt was observed only in a few farms, although the teliospores were found in the majority of the seed samples, resembles results from previous studies in similar countries, e.g., in Syria common bunt was present in all wheat fields, but at a low level [53]. Like common bunt, loose smut was only visually detected on few farms during the field survey. However, this may be explained by the fact that loose smut symptoms are visible at ear emergence, while the inspection in this study was performed later, during the flowering to dough stage. More than 20 other fungi species were isolated from the wheat samples. Some of these fungi are considered saprophytic, although they are known to cause damage in one of their life cycle phases, e.g., *Penicillium* spp. may cause decay in dry seeds [50]. In addition, some saprophytic fungi are actually known to cause toxicity, i.e., *Aspergillus* spp., *Rhizopus* spp. and *Chaetomium* spp. [14,54], and can become a serious health issue of human and animals from spoilt grain or feed [54]. Species from the genera *Aspergillus*, *Ulocladium*, *Epicoccum* and *Penicillium* are also known to be allergenic for human and animals [54]. In the present study, one of the main aims was to identify potential wheat pathogens. The following fungi species known to cause different diseases and damage in wheat were detected on grain samples: *Alternaria* spp., *Bipolaris sorokiniana*, *Stemphylium* spp., *Curvularia* spp., *Cladosporium* spp. and *Fusarium* spp. Thus, on the basis of both sustainability reasons in production and for food security reasons, we suggest that wheat breeding should focus on increased levels of resistance towards fungi and pathogens in the wheat seed.

*Alternaria* spp. was present in the majority of the samples analyzed and was also predominantly present in samples showing black point symptoms (~87%). Previous studies have commonly found *Alternaria* spp. in wheat seeds, but most have not observed any direct effect of this fungus on wheat quality [55]. Many studies have identified *Alternaria* spp. as one of the causes of black point [56,57,58] as in the present study. Presence of the disease may become the reason for the degradation of commercial quality of wheat grain [18,59]. Some studies have also indicated a decrease in germination capacity in wheat seeds with black point [60,61], although contradictory results have been found in other studies [58]. These contradictory results may be because different fungi can be present on wheat seeds showing black point, as found here. Khani et al. [17] found out that two major factors for black point development are susceptibility of variety and environment conditions during the grain development period, such as higher humidity and lower temperature. Further studies of Tajik wheat genotypes under the various agro-climatic conditions will allow farmers to select the most desired ones to achieve a decreased rate or even complete elimination of black point wheat grains.

Farmers surveyed in this study admitted that the first action they take once any diseases appear is to use pesticides. However, serious issues are known to be caused from the misuse of pesticides, both towards the environment with contamination of soils and food [62,63,64], but also on human health [65]. On the other side, the quality of the available pesticides in the market is also not well controlled in Tajikistan. Therefore, farmers in Tajikistan and similar countries are to be trained in how to use environmentally friendly approaches of diseases management in wheat production. The use of resistant varieties and best crop management practices, including the use of certified seeds and crop rotation practices, are among the most sustainable measures to be taken here [10].

Previous studies reported that a relationship was found between fungi on seeds and the protein quality of the grain [66]. As was discussed earlier, in this study a negative relationship was found between several of the fungi present on seeds and TOTE (Figure 4). TOTE is a known protein parameter correlating with grain protein concentration [25,67]. The same fungus correlated positively with %UPP, known to correlate with gluten strength [24,25]. However, there is generally a negative correlation between TOTE and %UPP [68], which might explain some of the correlations with fungi observed in the present study too. In general, the protein quality of grain samples in the present investigation did not show very high TOTE or %UPP values. Although there was rather high variation in the different protein factors between the samples, the values were of similar magnitude as for the standard variety Dragon, a Swedish spring wheat variety with normal grain protein concentration (11–13%) and rather weak gluten quality [31] that was used as a control in the SE-HPLC. The findings of relatively low protein content and gluten strength in Tajik wheat call for breeding activities to improve the yield and quality of local cultivars, which corresponds to breeding goals of the Tajik wheat breeding program to improve bread-making quality [21]. A high proportion of Tajik wheat breeding material holds the high molecular weight glutenin subunits 5 + 10 [69], and has a high grain protein concentration (unpublished results), offering good opportunities to achieve a better bread-making quality, thereby improving the food security. However, the issues with heterogeneity in the Tajik breeding material [69] and the use of saved seeds by farmers [13] need to be resolved for sustainable production of bread wheat in Tajikistan.

The present study evaluated possible effects of farm type, management and environmental factors such as elevation on the prevalence of seed-borne diseases and protein quality. Farm type was shown to have some impact on presence of fungi on wheat grain, with higher incidence of *Stemphillium* spp. and *Curvularia* spp. on grain from DF-type farms and of *B. sorokiniana* on grain from PC-type farms. Variations in the presence of diseases between farm types could possibly be related to differences in production conditions, availability of machinery and use of various inputs. In general, the larger farms surveyed had more organized wheat production than the smaller-scale farms, as was also reported earlier [10]. In particular, PC-type farmers knew the wheat variety they used, followed a crop rotation, used machinery for sowing and treated seed to control fungi. The positive correlation between knowledge of genotype grown and use of a crop rotation scheme corresponds with previous reports, as do the general findings on management in Tajik farms [10]. Thus, the present study confirms the need of farmer education for sustainable wheat production in Tajikistan to obtain food security.

Land elevation played a minor role for presence of seed-borne diseases and for protein quality. In general, the wheat genotype mainly determines the quality of the grain [26,70]. However, in this study, the effect of the genotype could not be properly evaluated since the farmers surveyed, especially on DF-type farms, had limited knowledge of the genotype they were actually growing. Such lack of knowledge as related to the genotype grown has in a previous study been correlated to a lack of success in wheat production [10]. Thus, human resource development (HRD) activities, such as training programs, are needed to educate farmers of all types in order to increase the success in wheat cultivation in Tajikistan. These should include formal training in schools, but also demonstration and dissemination of up-to-date information to farmers on sustainable crop production, including major diseases and weeds and opportunities, to improve the bread-making quality of wheat grain.

This study clearly showed that the health of homegrown wheat seeds in Tajikistan is rather poor, negatively effecting sustainable production and food security. Seed-borne diseases can be seen as a hidden threat to future sustainable crop production, with a negative impact on germination rate, number of normal seedlings and seed quality [15]. Previous studies have shown that use of certified seeds of tested and accepted varieties leads to higher yield and financial income [71]. A number of factors influence farmers’ willingness to use certified seeds, including knowledge level, financial capacity [71] and most importantly, availability and trust in the certified seeds [72]. Seed certification standards can help to control the spread of diseases, as certified seeds are usually free from pathogens causing seed-borne diseases [15], thereby preventing major grain losses [19]. Seed certification is currently not widely implemented throughout Tajikistan [73]. Developments of a proper seed certification and seed health testing system would significantly contribute to higher yielding and more sustainable wheat crop production in Tajikistan and in other developing countries.

## 5. Conclusions

The development of sustainable production of high-yielding, high-quality staple crops is important for developing countries and can be examined in wheat through the case of seed health status and protein composition. High levels of seed-borne diseases in the seeds together with low protein content and weak gluten were shown as two major issues for Tajik wheat production. High levels of fungal pathogens, including common bunt, were found on the wheat seeds despite the relatively low levels of visible common bunt symptoms in field. The farmers surveyed often did not know which wheat variety they were growing and used their own grain as seed for the next crop. There were no differences in the incidence of seed diseases and in grain baking quality between farm types, despite the fact that larger-scale farmers were better educated about wheat varieties. To improve wheat production and the sustainability of it in Tajikistan and other developing countries, certified seeds of resistant varieties should be made available and farmers should avoid using their own untested saved seeds and receive training on sustainable wheat production, including management of major diseases and weeds, and on opportunities to improve the bread-making quality of wheat grain.

Food security is a strategic objective of the Tajik government. Improving wheat production and the bread-making quality of wheat grain can be a major step in achieving this objective, since wheat-based bread is a staple food in Tajikistan and locally produced wheat varieties are currently a low priority for flour milling companies. The findings in this study regarding the health and quality of grain produced on different farm types can help decision makers plan and implement solutions, whereby wheat breeders develop new varieties and wheat growers understand and utilize the potential of modern breeding materials.

## Figures and Tables

**Table 1:** Name, location, coordinates, planted area and wheat varieties on the 21 farms in Tajikistan surveyed in this study, 2011–2012.

Farm ID	Location/District							Farm Type ^2^	Wheat Grown on Farm		
	Farm Name	Region	District	Jamoat ^1^ /Village	Coordinates		Elevation, Masl		Total Area, Ha	For Seed, Ha	Variety (s)
F1	Sidiq-bobo	Khatlon	Shahritus	Sayod	37°12.449′ N	68°08.598′ E	345	DF	0.8	0.8	Unknown
F2	Sh.Nazarov	Khatlon	Shahritus	Sayod	37°12.618′ N	68°08.450′ E	351	DF	5	0.4	Unknown
F3	Mahmadi Nuraliev	Khatlon	Qubodiyon	Faroghat	37°14.426′ N	68°10.132′ E	357	DF	1.5	0	Unknown
F4	Pulod-bobo	Khatlon	N.Khusrav	Oltinoy	37°17.803′ N	68°03.450′ E	401	DF	0.5	0.1	Unknown
F5	L.Murodov	DRS ^3^	Hisor	Dehqonobod/Chilchinor	38°30.230′ E	68°35.540′ E	753	PC	202	180	Lastochka, Sarvar, Tr. Khatti, Yasaul, Krasnodar 99
F6	Muminov	DRS	Hisor	Dehqonobod/Muminobod	38°30.220′ N	68°35.550′ E	761	DF	0.1	0.1	Krasnodar 99
F7	Dusti	DRS	Tursunzoda	Karotog	38°33.274′ N	68°16.547′ E	805	PC	170	31	Besribey, Moskvich
F8	Dahmarda	DRS	Fayzobod	Fayzobod	38°32.927′ N	69°19.478′ E	1178	DF	1	1	Steklovidnaya 24
F9	Junaydullo	Khatlon	Vakhsh	Tojikobod	37°46.651′ N	68°45.990′ E	419	DF	1.6	1	Unknown
F10	Mullo Mirzo	Khatlon	Vakhsh	Tojikobod	37°47.417′ N	68°46.330′ E	392	DF	3	3	Jayhun (uses for last 5 years)
F11	Tuychiboy	Sughd	Istaravshan	Javkandak	39°49.430′ N	69°03.350′ E	1270	DF	6	2	Umanka, Lastochka, Starshina
F12	Sayod	Sughd	Istaravshan	Javkandak	39°49.837′ N	69°03.389′ E	1252	DF	3	2	Krasnodar, Lastochka, Steklovidnaya 24
F13	Sughd TFI Branch	Sughd	Ghafurov	Isfisor/Aliev	40°12.454′ N	69°41.334′ E	358	TFI	24	12	Sadokat, Norman, Oriyoi. Different for research purposes
F14	Kattabek Juraev	Sughd	Konibodom	Ortikov/Shurkurgon	40°15.900′ N	70°22.601′ E	355	DF	2.85	2.85	Starshina
F15	Mukarramov	Sughd	Isfara	Kulkand	40°09.366′ N	70°41.830′ E	800	PC	135	105	Starshina, Yasaul, Gratsiya, Pervitsa, etc. Mostly Russian varieties
F16	Kulkand	Sughd	Isfara	Kulkand	40°09.337′ N	70°41.600′ E	799	DF	2	1	Gratsiya, Starshina, Russian varieties
F17	T. Kattaev	Sughd	Isfara	Chilagzi	40°09.870′ N	70°44.421′ E	826	PC	129	10	Sarvar, Starshina, Ziroat 70
F18	Salom	Khatlon	Vose	Salom	37°55.769′ N	69°43.897′ E	567	PC	54	12	Jayhun
F19	Khatlon TFI branch of FI	Khatlon	Bokhtar	Nikhi	37°51.583′ N	68°47.092′ E	431	TFI	6	1	Different for research purposes: Sadokat, Alex, Besribey, etc.
F20	Hamadoni	Khatlon	Kulob	Ziraki	37°57.150′ N	69°47.489′ E	600	PC	350	120	Yasaul, Jayhun, Besribey
F21	Muminobod	Khatlon	Muminobod	Gofilobod	38°17.714′ N	70°05.463′ E	1334	DF	6	1	Besribey

^1^ Local administrative division consisting of one or more villages. ^2^ DF—*dehkan* farm; PC—production cooperative; TFI—Tajik Farming Institute ^3^ DRS—District of Republican Subordination under the direct rule of central government.

**Table 2:** Presence of *Tilletia* spp. (as frequency of infection) on each farm surveyed over the two study years.

Farm ID	*T. laevis*	*T. tritici*
F1	100 a	37.5 abc
F2	100 a	0 c
F3	100 a	25 bc
F4	100 a	50 ab
F5	0 d	0 c
F6	25 bcd	0 c
F7	100 a	0 c
F8	100 a	37.5 abc
F9	62.5 ab	0 c
F10	12.5 cd	0 c
F11	100 a	75 a
F12	100 a	62.5 ab
F13	100 a	0 c
F14	25 bcd	0 c
F15	0 d	0 c
F16	12.5 cd	0 c
F17	25 bcd	0 c
F18	100 a	0 c
F19	100 a	0 c
F20	50 bc	0 c
F21	100 a	0 c
Farms	***	***
Years	n/s	**
Test replicate	n/s	n/s

Values within a column with different letters differ significantly (n/s = not significant; ** = *p* < 0.01; *** = *p* < 0.001).

**Table 3:** Prevalence of major fungi (expressed as %, frequency of infection) identified in grain samples from the farms surveyed.

Farm ID	*Alternaria* spp.		*Bipolaris sorokiniana*		*Stemphylium* spp.		*Curvularia* spp.		*Cladosporium* spp.		*Fusarium* spp.		Other Fungi (Mainly Saprophytes)	
	2011	2012	2011	2012	2011	2012	2011	2012	2011	2012	2011	2012	2011	2012
F1	92.3	86.9	0.0	0.8	3.8	0.8	0.0	2.3	0.0	0.0	0.0	0.0	3.8	9.2
F2	90.0	84.0	2.3	0.0	0.0	2.3	1.5	1.5	0.0	0.0	0.0	0.8	6.2	11.5
F3	55.0	82.4	0.0	0.8	0.0	0.8	0.0	0.0	0.0	1.6	0.0	0.0	45.0	14.4
F4	85.4	80.6	0.0	2.2	3.1	0.0	0.8	0.0	0.0	0.0	0.0	1.5	10.8	15.7
F5	86.2	84.6	1.5	2.3	1.5	0.0	3.1	3.1	0.8	0.0	0.0	2.3	6.9	7.7
F6	79.2	66.7	2.3	6.0	6.2	2.6	0.0	0.0	1.5	0.0	0.0	2.6	10.8	22.2
F7	23.2	31.2	0.0	2.8	1.4	2.1	0.0	0.0	0.0	1.4	0.0	0.0	75.4	62.4
F8	65.3	63.7	0.8	1.6	2.4	1.6	0.8	0.8	0.0	2.4	0.0	0.0	30.6	29.8
F9	90.0	83.8	0.0	0.0	1.5	1.5	0.0	0.8	0.0	0.0	0.0	1.5	8.5	12.3
F10	83.8	80.6	0.0	0.7	0.8	4.5	0.0	1.5	0.0	0.0	1.5	3.0	13.8	9.7
F11	64.6	41.0	0.0	1.0	0.8	0.0	1.5	0.0	5.4	1.9	1.5	0.0	26.2	56.2
F12	51.2	43.5	0.0	0.0	0.0	1.9	0.0	0.9	4.1	1.9	0.0	0.0	44.6	51.9
F13	73.3	37.2	0.0	0.0	0.0	5.8	0.0	0.0	1.5	0.0	0.0	0.0	25.2	57.0
F14	47.9	17.4	0.0	0.0	2.1	0.0	0.0	0.0	0.7	0.0	0.0	0.0	49.3	82.6
F15	96.2	87.9	0.0	0.0	0.8	0.0	0.8	1.5	0.0	0.0	1.5	3.8	0.8	6.8
F16	97.7	91.5	0.0	0.8	0.8	0.0	0.0	0.0	0.0	0.0	0.8	4.6	0.8	3.1
F17	87.8	87.2	0.0	0.0	1.5	1.7	2.3	2.6	0.8	0.0	0.8	0.9	6.9	7.7
F18	63.8	68.1	3.1	11.1	1.5	5.2	3.1	5.2	0.0	0.0	0.0	3.0	28.5	7.4
F19	83.3	64.7	0.0	1.5	3.0	0.0	3.0	0.8	0.0	0.0	0.0	1.5	10.6	31.6
F20	30.7	61.1	0.7	14.5	0.0	0.0	0.7	4.6	0.7	12.2	0.0	0.0	67.2	7.6
F21	68.9	77.8	4.1	6.8	0.0	3.4	0.8	1.7	0.0	0.9	0.0	0.9	26.2	8.5

**Table 4:** Frequency of wheat grain infection with major seed-borne fungi categorized according to farm type (DF = *dehkan* farm; PC = production cooperative; TFI = Tajik Farming Institute farm).

Farm Types	Major Isolated Fungi					
	*Alternaria* spp.	*Bipolaris Sorokiniana*	*Stemphylium* spp.	*Curvularia* spp.	*Cladosporium* spp.	*Fusarium* spp.
DF	91 a	1.4 b	2.0 a	0.7 b	1.0 a	0.9 a
PC	87 a	4.0 a	1.7 b	2.9 a	1.8 a	1.3 a
TFI	84 a	0.5 b	2.8 a	1.3 b	0.5 a	0.5 a
Significance (*p*)	0.366	0.000	0.388	0.000	0.120	0.337

Tukey’s post hoc test: means that do not share a letter are significantly different (*p* < 0.05).

**Table 5:** Frequency of infection by black point in wheat grain samples from the 21 Tajik farms surveyed in two study years.

Farm ID	2011	2012
F1	6.8	2.4
F2	1.6	1.5
F3	0.0	3.6
F4	1.9	27.8
F5	1.7	3.4
F6	5.4	3.6
F7	0.0	0.0
F8	0.6	0.6
F9	2.0	1.2
F10	5.3	5.0
F11	1.2	0.0
F12	0.0	0.0
F13	0.7	0.7
F14	3.7	0.0
F15	1.4	3.5
F16	3.7	0.7
F17	3.8	0.6
F18	0.0	1.9
F19	1.9	2.6
F20	0.0	0.6
F21	0.0	2.6

^ns^ year = *p* = 0.47, F4 significantly higher than other farms. Colors: green boxes with no black point infection and red boxes with higher infection rates.

**Table 6:** Relative amount of various protein fractions determined by SE-HPLC in wheat samples from 21 Tajik farms surveyed, and significance levels for protein factors as related to farm type and study years based on analysis of variance.

Farm ID	TOTE (10^7^)	TOTU (10^7^)	%UPP	%LargeUPP
Dragon (c) ^a^	11.7 abc	4.6 de	46.0 bcde	52.8 cde
F1	10.3 bc	4.8 cde	45.9 cde	54.2 cde
F2	12.7 ab	5.8 bcd	45.9 cde	52.8 cde
F3	12.1 abc	6.4 ab	50.8 abcde	59.0 abcde
F4	10.6 bc	5.4 bcd	49.7 abcde	57.7 abcde
F5	10.1 bc	5.7 bcd	55.1 ab	64.5 ab
F6	10.8 abc	4.8 cde	46.8 bcde	55.9 bcde
F7	12.1 abc	5.5 bcd	47.6 bcde	56.7 bcde
F8	10.9 abc	5.0 cde	45.6 cde	52.4 cde
F9	9.8 bc	6.5 ab	58.1 a	66.1 a
F10	10.0 bc	4.8 cde	47.4 bcde	55.0 cde
F11	9.3 c	4.6 de	51.7 abcde	60.6 abcd
F12	10.2 bc	4.0 e	44.4 e	51.9 de
F13	12.9 ab	5.5 bcd	45.1 de	54.6 cde
F14	11.4 abc	7.2 a	53.7 abcd	59.9 abcd
F15	13.9 a	5.9 abcd	44.3 e	50.5 e
F16	12.8 ab	5.8 bcd	45.1 de	50.4 e
F17	12.3 abc	5.7 bcd	46.9 bcde	53.5 cde
F18	10.1 bc	5.7 bcd	54.1 abc	61.5 abc
F19	11.0 abc	5.6 bcd	50.5 abcde	57.4 abcde
F20	9.4 c	5.0 cde	50.0 abcde	57.0 abcde
F21	10.7 bc	6.0 abc	52.0 abcde	60.4 abcd
Farms	*p* > 0.001 ***	*p* > 0.001 ***	*p* > 0.001 ***	*p* > 0.001 ***
Year	*p* > 0.001 ***	*p* = 0.039 *	*p* = 0.011 *	*p* = 0.349 ^ns^
Test replicate	*p* = 0.978 ^ns^	*p* = 0.936 ^ns^	*p* = 0.937 ^ns^	*p* = 0.866 ^ns^

^a^ The Swedish variety Dragon was used as the control in SE-HPLC analyses. Values within columns with different letters differ significantly at *p* < 0.05 applying Tukey’s post hoc test; ns = not significant: *, *** = significant at *p* < 0.05, 0.01, 0.005. TOTE = Total SDS-extractable proteins; TOTU = Total SDS-unextractable proteins; %UPP = Percentage of total unextractable polymeric proteins in total polymeric proteins; %LargeUPP = Percentage of large unextractable polymeric proteins in total large polymeric proteins.

## Data Availability

Not Applicable.

## Supplementary Materials

The following are available online at https://www.mdpi.com/article/10.3390/ijerph18115751/s1, Table S1: Questionnaire; Table S2: General wheat management practices; Table S3: Summary of disease incidence and major constraints to wheat production in the fields surveyed.
