# Supplementary material for: Sustainable Wheat Production and Food Security of Domestic Wheat in Tajikistan: Implications of Seed Health and Protein Quality
Source: Int J Environ Res Public Health. 2021 May 27;18(11):5751. doi: 10.3390/ijerph18115751 (PMC8198249; doi:10.3390/ijerph18115751)
Supplement: Supplementary file 1 [file ijerph-18-05751-s001.zip › ijerph-1206842-SI1.pdf]

## Supplementary material

**Table S2.**

**General wheat management practices**

| ID | Variety name | Growing purpose | Planting time | Planting method, both years | Seed Treatment | Seed rate (kg/ha) | Pesticide use in the field | Weed control | Irrigation type | Previous crop (rotation) | | Fertiliser | | |
|---|---|---|---|---|---|---|---|---|---|---|---|---|---|---|
| | | | | | | | | | | 2011 | 2012 | Type | Amount (kg/ha) | Applied time |
| F1 | Unknown | grain | Oct.-Nov. | hand broadcast | no | 180-200 | No | No | Irrigated | watermelon | cotton | N | 90-100 | Tillering / Earing |
| F2 | Unknown | grain | Oct.-Nov. | hand broadcast | no | 180-200 | No | No | Irrigated | watermelon | cotton | N | 100 | Tillering / Earing |
| F3 | Unknown | grain | Oct.-Nov. | hand broadcast | no | 180-200 | No | No | Irrigated | cotton | cotton | N | 120 | Booting |
| F4 | Unknown | grain | Oct.-Nov. | hand broadcast | no | 180-200 | No | No | Irrigated | fallow | cotton | N | 30-50 | Tillering |
| F5 | Lastochka | seed/grain | Oct.-Nov. | drill | Dividend / Vitavax | 180-200 | No | Hand weeding | Irrigated | cotton | maize | N | 120 | Booting |
| F6 | Krasnodar 99 | grain | Oct.-Nov. | hand broadcast | no | 180-200 | No | Hand weeding | Irrigated | maize | potato | N | 30-50 | Booting |
| F7 | Besribey | seed/grain | Nov. | drill | Vitavax | 200-220 | No | No | Irrigated | cotton | maize | N | 120 | Booting / Earing |
| F8 | Steklovidnaya 24 | grain | Oct. | hand broadcast | no | 180-200 | No | No | Rainfed | chick pea | wheat | N | 120 | Booting |
| F9 | Unknown | grain | Nov. | hand broadcast | no | 180-200 | No | Hand weeding | Irrigated | watermelon | wheat | N | 100 | Tillering / Earing |
| F10 | Jayhun | grain | Oct. | drill | no | 180-200 | No | Hand weeding | Irrigated | cotton | cotton | N | 120 | Earing |
| F11 | Umanka | grain | Sep.-Oct. | broadcast | no | 220-240 | No | Hand weeding | Irrigated | potato | potato | N | 60 | Earing |
| F12 | Steklovidnaya 24 | grain | Oct. | hand broadcast | no | 220-240 | No | Herbicide (Gezagard) | Irrigated | potato | potato | N | 90 | Earing |

| | | | | | | | | | | | | | | |
|---|---|---|---|---|---|---|---|---|---|---|---|---|---|---|
| F13 | Sadokat | seed/grain | Nov.-Dec. | hand broadcast | TMTD | 180-200 | No | No | Irrigated | rape for fodder | maize | N | 120 | Booting / Earing |
| F14 | Starshina | seed/grain | Sep.-Oct. | hand broadcast | TMTD / Vitavax | 200-220 | No | hand weeding | Irrigated | cotton | cotton | N | 120 | Booting / Earing |
| F15 | Yasaul | seed/grain | Sep.-Oct. | drill | Raksill | 200-220 | No | hand weeding / herbicide (Granstar) | Irrigated | tomato | watermelo n | N | 150 | Booting / Earing |
| F16 | Gratsiya | seed/grain | Oct. | drill | Raksill | 200-220 | No | hand weeding / herbicide (Granstar) | Irrigated | tomato | tomato | N | 150 | Booting / Earing |
| F17 | Starshina | seed/grain | Oct. | drill | no | 200-220 | No | No | Irrigated | maize | tomato | N | 150-180 | Tillering / Earing |
| F18 | Jayhun | seed/grain | Oct.-Nov. | drill | no | 200 | No | No | Irrigated | cotton | wheat | N | 110 | Booting |
| F19 | L115 | seed/grain | Oct.-Nov. | drill | no | 200 | No | No | Irrigated | cotton | barley | N | 160 | Tillering / Booting |
| F20 | Yasaul | seed/grain | Oct.-Nov. | drill | Dividend | 200 | No | No | Irrigated | cotton | cotton | N | 150 | Booting / Earing |
| F21 | Besribey | grain | Oct. | hand broadcast | no | 200 | No | No | Rainfed | potato | potato | N | 100 | Booting |

**Table S3.**

**Summary of disease incidence and major constraints to wheat production in the fields surveyed**

| Sample ID | Growing stage | | Loose smut | | Common bunt | | Other diseases | | Weed density | |
|---|---|---|---|---|---|---|---|---|---|---|
| | 2011 | 2012 | 2011 | 2012 | 2011 | 2012 | 2011 | 2012 | 2011 | 2012 |
| F1 | milk | flowering to milk stage | Not recorded | Not recorded | Not recorded | Not recorded | Septoria, YR (2-3%) | Septoria, TS | high | medium |
| F2 | milk | floweing to milk stage | Not recorded | Not recorded | Not recorded | Not recorded | LS | | medium | high |
| F3 | milk | floweing to milk stage | Not recorded | Not recorded | >50% | Not recorded | LS | | medium | medium |
| F4 | milk | milk | Not recorded | Not recorded | <25% | Not recorded | LS | TS | medium | medium |
| F5 | flowering | flowering, beginning of milk | Not recorded | Not recorded | Not recorded | Not recorded | YR (2-3%), TS and other LSs | | medium | low |
| F6 | flowering | dough | Not recorded | Not recorded | Not recorded | Not recorded | YR and TS | TS, PM | medium | medium |
| F7 | flowering | flowering | <1% | Not recorded | Not recorded | Not recorded | TS | TS | medium | medium |
| F8 | not even, some in heading some are flowering | flowering | Not recorded | Not recorded | Not recorded | Not recorded | TS, YR, PM | | high | high |
| F9 | dough stage | flowerning-early milk | Not recorded | Not recorded | Not recorded | Not recorded | Plant were too dry | | high | high |
| F10 | milk | flow | Not recorded | <1% | Not recorded | Not recorded | TS | TS | medium | medium |
| F11 | flowering | flowerning-early milk | Not recorded | Not recorded | Not recorded | Not recorded | LS | PM | low | medium |
| F12 | flowering | early flowering | Not recorded | Not recorded | Not recorded | Not recorded | LS | | low | high |

| | | | | | | | | | | |
|---|---|---|---|---|---|---|---|---|---|---|
| F13 | milk | milk to dough | Not recorded | Very low | 5 % | Not recorded | YR, LR | LR, | high | medium |
| F14 | milk-dough | dough | Not recorded | Not recorded | Not recorded | Not recorded | | | high | medium |
| F15 | milk | early milk | Not recorded | Not recorded | Not recorded | Not recorded | TS and few rust | TS | high | medium |
| F16 | milk | milk | Not recorded | Not recorded | Not recorded | Not recorded | TS | TS | high | low |
| F17 | milk | milk | Not recorded | Not recorded | Not recorded | Not recorded | TS | YR (v.low). LR (v.low) | high | low |
| F18 | matured | dough | Not recorded | Very low | Not recorded | Not recorded | LS | TS, root rot | medium | high |
| F19 | dough | milk | Not recorded | Not recorded | Not recorded | Not recorded | TS and YR (few) | | high | high |
| F20 | dough | milk | Not recorded | Not recorded | Not recorded | Not recorded | LS | TS, root rot | med | high |
| F21 | milk-dough | milk | Not recorded | Not recorded | Not recorded | Not recorded | LS and TS | Septoria, TS | high | high |

Note: YR-yellow rust; TS-Tan spot; LS-leaf spot; PM-powdery mildew;
