# Supplementary material for: Sustainable Wheat Production and Food Security of Domestic Wheat in Tajikistan: Implications of Seed Health and Protein Quality
Source: Int J Environ Res Public Health. 2021 May 27;18(11):5751. doi: 10.3390/ijerph18115751 (PMC8198249; doi:10.3390/ijerph18115751)
Supplement: Supplementary file 1 [file ijerph-18-05751-s001.zip › ijerph-1206842-SI2.pdf]

QUESTIONNAIRE
Wheat seed-borne diseases survey in Tajikistan

…………………………	……………………	…………………………
survey date	round	conducted by

General Information about Farm

Farm name:

Address: ………………………………….. (region/location) ……………………………………………….. (postal address)

Contacts: ………………………………….. (tel./fax/mob.) ……………………………………………….. (e-mail/other)

Representative: ………………….. (name) ………………… (contacts: tel./mob.) ………………………………… (position)

Wheat in the farm: ………………….. (total wheat area (ha)) ………………… (for seed prod. (ha)) ………………………………… (varieties)

Field and crop management information

Latitude: N S ……………..…. Longitude: E W ……….……... Altitude (m): ……………..…..

Field: …………………….. (size (ha)) …………………………………. (previous crop) ………………………………….. (variety)

Planting: …………………….. (planting date) …………………… (method (row/broadcast)) …………………… (seed treatment (dose)) …………………… (seed rate (kg/ha))

Crop type: Food: ☐ Seed: ☐ Feed: ☐ Other: …………………………………

Management:

Fertilizer: …………………….. (type (N, P, K)) …………………………………. (rate for each type (kg/ha, pure)) ………………………………….. (time)

Pesticide: …………………….. (type (ins., fung.)) …………………………………. (dose (for each, l/ha-pure)) ………………………………….. (time)

Weed control: hand weeding / chemical control / other ……………………………………………

Irrigation: irrigated / rain fed

Irrigation time: …………………….. (irrigation type) …………………………………. (dates of 1st, 2nd and following) ………………………………….. (date of last irrigation/rain)

Field survey

Observation: ………………………………….. (crop view) ………………………………… (growth stage) …………………… (lodging)

Weed: ………………………………….. (density (high, med., low)) ……………………………………………………………. (predominant species)

Diseases:

Loose smut: …………………………………………………………………………..

Common bunt: …………………………………………………………………………..

Other diseases: …………………………………………………………………………..

(name and incidence …………………………………………………………………………..

(occurence) level) …………………………………………………………………………..

Notes: ……………………………………………………………………………………………

……………………………………………………………………………………………

……………………………………………………………………………………………
